# Cancer killers in the human gut microbiota: diverse phylogeny and broad spectra

**DOI:** 10.18632/oncotarget.17319

**Published:** 2017-04-21

**Authors:** Yu-Jie Zhou, Dan-Dan Zhao, Huidi Liu, Hao-Ting Chen, Jia-Jing Li, Xiao-Qin Mu, Zheng Liu, Xia Li, Le Tang, Zhan-Yi Zhao, Ji-Heng Wu, Yu-Xuan Cai, Ya-Zhuo Huang, Peng-Ge Wang, Yi-Yue Jia, Pei-Qiang Liang, Xue Peng, Si-Yu Chen, Zhi-Lin Yue, Xin-Yuan Yuan, Tammy Lu, Bing-Qing Yao, Yong-Guo Li, Gui-Rong Liu, Shu-Lin Liu

**Affiliations:** ^1^ Systemomics Center, College of Pharmacy, and Genomics Research Center, State-Province Key Laboratories of Biomedicine-Pharmaceutics of China, Harbin Medical University, Harbin, China; ^2^ HMU-UCFM Centre for Infection and Genomics, Harbin Medical University, Harbin, China; ^3^ Colorectal Surgery Department, Cancer Hospital, Chinese Academy of Medical Sciences & Peking Union Medical College, Beijing, China; ^4^ Department of Infectious Diseases, The First Affiliated Hospital, Harbin Medical University, Harbin, China; ^5^ Department of Microbiology, Immunology and Infectious Diseases, University of Calgary, Calgary, Canada; ^6^ Current affiliation: Department of Ecosystems and Public Health, University of Calgary, Calgary, Canada; ^7^ Current affiliation: Life Sciences, Queen's University, Kingston, Canada; ^8^ Current affiliation: Biomedical Science, University of Calgary, Calgary, Canada

**Keywords:** cancer, malignancy killer, Actinobacteria, gut microbiota, leukemia

## Abstract

Cancer as a large group of complex diseases is believed to result from the interactions of numerous genetic and environmental factors but may develop in people without any known genetic or environmental risks, suggesting the existence of other powerful factors to influence the carcinogenesis process. Much attention has been focused recently on particular members of the intestinal microbiota for their potential roles in promoting carcinogenesis. Here we report the identification and characterization of intestinal bacteria that exhibited potent anti-malignancy activities on a broad range of solid cancers and leukemia. We collected fecal specimens from healthy individuals of different age groups (preschool children and university students), inspected their effects on cancer cells, and obtained bacteria with potent anti-malignancy activities. The bacteria mostly belonged to Actinobacteria but also included lineages of other phyla such as Proteobacteria and Firmicutes. In animal cancer models, sterile culture supernatant from the bacteria highly effectively inhibited tumor growth. Remarkably, intra-tumor administration of the bacterial products prevented metastasis and even cleared cancer cells at remote locations from the tumor site. This work demonstrates the prevalent existence of potent malignancy-killers in the human intestinal microbiota, which may routinely clear malignant cells from the body before they form cancers.

## INTRODUCTION

Cancer includes a large group of malignancies that are believed to occur as a combined consequence of genetic and environmental factors, since many genetic traits have been demonstrated to suppress or promote carcinogenesis as cancer suppressors or oncogenes, and environmental factors may help prevent or promote cancers [1–3]. But individuals born to a family that has high genetic risks to cancers do not necessarily develop cancers in their lifetime. Conversely, many cancer patients do not have a high-risk familial genetic background nor contacts to known harmful environmental factors like tobacco, industrial pollutants, etc.

A recent study shows that the cancer risk is strongly correlated with the number of cell divisions [4]. Based on this finding, the authors explain that the majority of cancerous variation is due to random mutations arising during DNA replication in stem cells, which the authors call “bad luck”. Indeed, it is not hard to understand that the more cycles of DNA replication a cell undergoes, the more random errors it will accumulate and hence the more chances a malignant mutation appears, leading to cancer as a result. Therefore, it is a quite reasonable explanation to a fact recognized for over a century that some tissue types give rise to cancers much more frequently than other types due to their greater turnover frequencies. However, it does not explain why the majority of people in the general populations, especially those under various genetic or environmental risks to cancer, do not develop cancer for their eighty years or even longer lifetime in any tissue. This question has led us to believe that there must be so far an unknown kind of factors, non-genetic and non-environmental in the traditional meaning, in addition to immunity, that constantly work in the healthy human body to clear malignant cells timely to prevent them from forming cancers.

Previously, we investigated the intestinal microbiota for its potential associations with human health and isolated bacteria that produce the anti-cancer agent enterodiol or secoisolariciresinol [5–9] by converting precursors contained in plants such as defatted flaxseeds through biotransformation [10–13]. Results of these studies prompted us to postulate that the intestinal microbiota might also contain microbes that attack malignant cells more directly, e.g., using *de novo* chemical substances, and lack of such microbial malignancy-killers may make people more vulnerable to cancer-causing factors. In this study, we screened the intestinal microbiota of two representative populations and isolated bacterial strains that produced potent anti-cancer substances. The isolated bacterial malignancy-killers were diverse by phylogeny and active against broad ranges of cancer types *in vitro* and *in vivo*. This finding demonstrates the existence of another potent anti-cancer system in the human body in addition to the cancer suppression and immune systems and reiterates the importance to keep our intestinal microbiota undisturbed by avoiding unhealthy life styles such as unbalanced dietary structure or smoking and inappropriate medical procedures such as misuse of antimicrobials.

## RESULTS

### Detection of anti-malignancy activities in bulk fecal specimens

**Figure 1:**
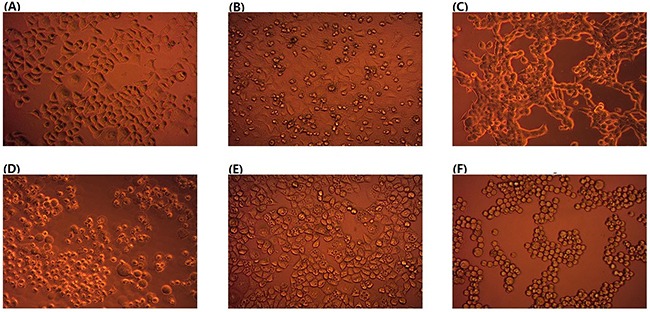
Effects of human fecal supernatant on HeLa cells **(A)**, control without fecal supernatant; **(B) through (F)**, fecal supernatant from five different individuals (Magnification, 200 X).

We first screened the fecal specimens collected from preschool children and young adults (See Materials and Methods) for their inhibiting effects on malignant cells. For this, we mixed the fresh fecal specimens with equal volume of sterile water before centrifugation and then added a 5 μl aliquot of the filtered fecal supernatant to 100 μl of cancer cell cultures. We inspected the effects under a microscope at 6 hours intervals. From many of the fecal specimens, we detected damaging effects on cancer cells, which led to various morphological changes such as cells exhibiting pyknosis, forming vacuoles and turning round (Figure 1).

Of remarkable significance, there was a strong tendency that preschool children had a much higher percentage of individuals exhibiting the anti-cancer effects than young adults (Table 1). Although the difference was statistically significant, we believed that this finding would need to be validated by larger scales of screening or more sensitive methods for the detection. Alternatively, detection of the producers of the anti-cancer substances would yield more direct evidence for the existence of the malignancy-killers and can also provide the materials for further studies or applications.

**Table 1:** Percentages of individuals whose fecal specimens exhibit positive anti-cancer activities in different age populations

Age group	No. samples	No. positive samples	Percentage of positive samples	
3-7 years	100	59	59.00%	
18-39 years	113	12	10.62%	**P<0.001
Total	213	71	33.33%	

Note: The *P* value indicates comparison between different age groups with the chi-square test by SPSS.

### Isolation of malignancy-killing bacterial strains

To isolate and identify the microbes that are responsible for the detected anti-malignancy activities, we spread diluted fecal specimens onto agar plates (see Materials and Methods), picked single colonies and grew the bacteria in liquid medium for up to 168 hours before inspecting their effects on cancer cells. From the collected 213 fecal specimens, we obtained 76 strains that showed various anti-malignancy effects. Supplementary Table 1 shows the detailed information about the participants and the anti-malignancy bacterial strains isolated from them. Notably, we isolated cancer-killing bacteria not only from fecal specimens tested positive but also from those tested negative in the supernatant for anti-malignancy activities, demonstrating that the final expression of the cancer-killing effects of these bacteria might be the net result of modulations of multiple biological events and of interactions among different microbes in the gut.

**Figure 2:**
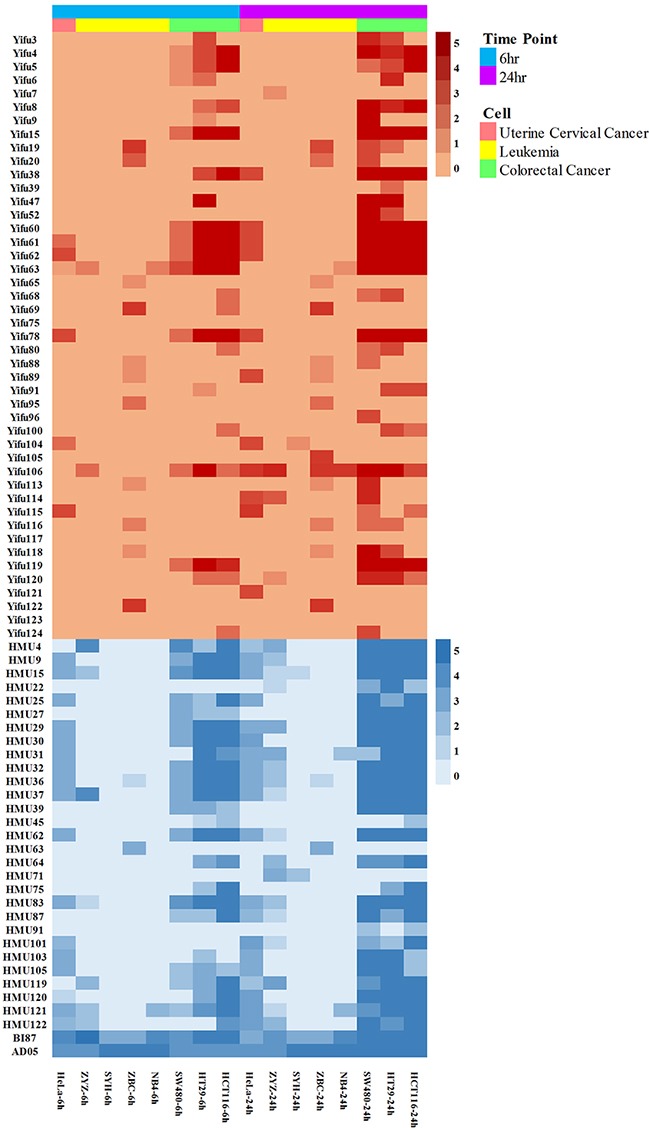
Anti-cancer effects of the bacterial strains against cervical cancer, leukemia and colorectal cancer

Different strains exhibited various anticancer activities against solid malignant tumors, including cervical cancer cell line HeLa, colorectal cancer cell lines SW480, HCT116 and HT29, and hematological malignancies, including human acute promyelocytic leukemia M3 cell line NB4, a primary culture of freshly isolated acute monocytic leukemia M5 strain ZYZ and chronic lymphocytic leukemia strain ZBC. Obviously, a majority of the 76 bacterial strains could kill solid tumors, especially the colorectal cancer, with much higher efficacy than they kill leukemia cells; however, we did find strains that killed leukemia cells highly effectively (Figure 2).

### Phylogenetic distribution of the anti-malignancy bacteria

**Figure 3:**
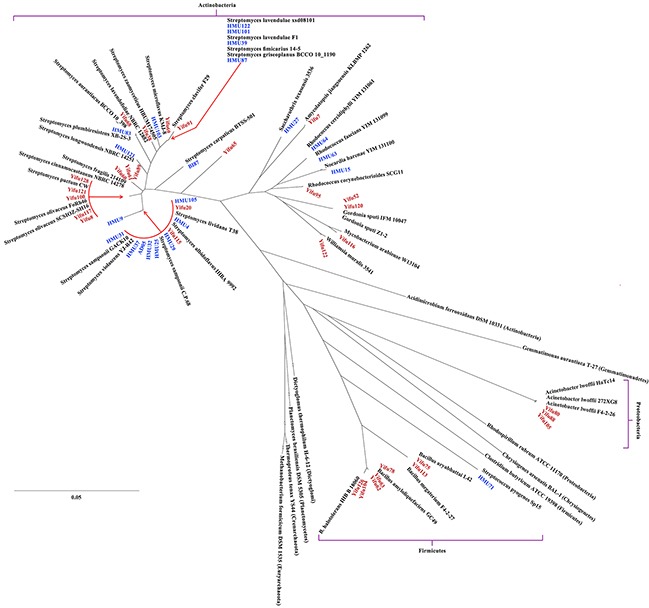
Phylogenetic distribution of the anti-cancer bacteria isolated in this study Two archeal strains and strains of several representative bacterial phyla were included to illustrate the relative phylogenetic positions of the anti-cancer bacteria. Red, strains isolated from preschool children; blue, strains isolated from young adults.

**Figure 4:**
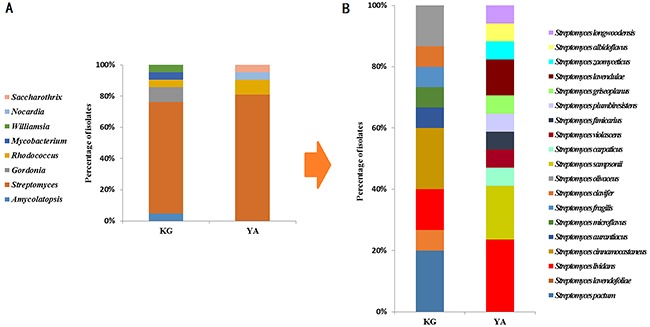
The relative abundance of human gut actinomycetes from kindergarten children and young adults **(A)** comparison at the genus level of Actinobacteria; and **(B)** comparison at the species level of *Streptomyces*.

To identify and characterize the bacteria that produced anti-malignancy effects, we shortlisted fifty-three strains for identification by 16S rDNA sequencing and compared them with known bacteria in the NCBI database (https://www.ncbi.nlm.nih.gov/genome/). Phylogenetic analyses showed that they mostly belonged to Actinobacteria but also contained lineages belonging to other phyla, including Proteobacteria and Firmicutes (Table 2), which are distantly related from one another in evolution (Supplementary Figure 1). Within the Actinobacteria phylum, the phylogenetic distribution of the bacteria was also broad (Supplementary Figure 2), with bacteria in the *Streptomyces* genus being most abundant in both the kindergarten (71.4%) and young adult (81.0%) groups (Figure 3). Another common genus to both groups was *Rhodococcus*. Overall, phylogenetic diversity of the anti-malignancy bacteria was higher in the kindergarten group than the young adult group on the genus level (Figure 4A). Some rare species, such as those of *Amycolatopsis jiangsuensis* and *Gordonia sputi*, were isolated from the kindergarten but not the young adult group. Within the genus of *Streptomyces*, the species were so diverse between the two groups that only one species, *Streptomyces lividans*, was found to be common to both of them (Figure 4B). These results suggest that the composition of the human intestinal microbiota may vary with age as previously reported [17], but more refined correlations, especially regarding the abundance changes of cancer-killing bacteria with age, would require larger sample sizes in the investigation to establish.

**Table 2:** Phylogenetic positions of the bacterial strains with strong anti-cancer activities

Strain	Sample ID	Known bacteria with closest relatedness (Accession numbers)	Ident	Phylum
Yifu7	KG-JZH	*Amycolatopsis jiangsuensis* KLBMP 1262 (NR_109638.1)	99%	Actinobacteria
Yifu8	KG-JZH	*Streptomyces pactum* CW (KX372557.1)	100%	Actinobacteria
Yifu20	KG-HYX	*Streptomyces lividans* T38 (KU317912.1)	100%	Actinobacteria
Yifu38	KG-SR	*Streptomyces lavendofoliae* NBRC 12882 (NR_112318.1)	100%	Actinobacteria
Yifu52	KG-HBY	*Gordonia sputi* IFM 10047 (FJ536305.1)	100%	Actinobacteria
Yifu60	KG-ZYC	*Streptomyces cinnamocastaneus* NBRC 14278 (AB184588.1)	99%	Actinobacteria
Yifu61	KG-ZYC	*Streptomyces cinnamocastaneus* NBRC 14278 (AB184588.1)	99%	Actinobacteria
Yifu62	KG-ZYC	*Brevibacterium halotolerans* IHB B 18060 (KU605238.1)	99%	Firmicutes
Yifu63	KG-ZYC	*Bacillus amyloliquefaciens* GC49 (KF158228.1)	100%	Firmicutes
Yifu65	KG-WZH	*Streptomyces cinnamocastaneus* NBRC 14278 (AB184588.1)	95%	Actinobacteria
Yifu68	KG-JYX	*Streptomyces aurantiacus* BCCO 10_398 (KP718492.1)	99%	Actinobacteria
Yifu69	KG-JYX	*Streptomyces microflavus* KMJ-8 (KJ020690.1)	99%	Actinobacteria
Yifu75	KG-ZJH	*Bacillus aryabhattai* L42 (KU179345.1)	100%	Firmicutes
Yifu78	KG-SJX	*Bacillus amyloliquefaciens* GC49 (KF158228.1)	100%	Firmicutes
Yifu80	KG-PJY	*Acinetobacter lwoffii* F4-2-26 (KX350033.1)	99%	Proteobacteria
Yifu88	KG-CBC	*Acinetobacter lwoffii* HaTc14 (KX150815.1)	99%	Proteobacteria
Yifu89	KG-CBC	*Streptomyces fragilis* 214109 (JN180226.1)	99%	Actinobacteria
Yifu91	KG-WX	*Streptomyces clavifer* F29 (KU324446.1)	100%	Actinobacteria
Yifu95	KG-WJZ	*Rhodococcus corynebacterioides* SCG11 (KU995335.1)	99%	Actinobacteria
Yifu100	KG-YQT	*Streptomyces olivaceus* SCSIOZ-SH16 (KC747484.1)	100%	Actinobacteria
Yifu104	KG-TZ	*Brevibacterium halotolerans* IHB B 18060 (KU605238.1)	100%	Firmicutes
Yifu105	KG-XZH	*Acinetobacter lwoffii* 272XG8 (KF818627.1)	99%	Proteobacteria
Yifu113	KG-YPY	*Bacillus megaterium* F4-2-27 (KX350034.1)	100%	Firmicutes
Yifu115	KG-XWR	*Streptomyces lividans* T38 (KU317912.1)	99%	Actinobacteria
Yifu116	KG-XWR	*Mycobacterium arabiense* W13104 (KJ676965.1)	99%	Actinobacteria
Yifu117	KG-LEQ	*Streptomyces pactum* CW (KX372557.1)	100%	Actinobacteria
Yifu120	KG-ZJH	*Gordonia sputi* Z1-2 (KJ571101.1)	99%	Actinobacteria
Yifu121	KG-WX	*Streptomyces olivaceus* FoRh46 (KM370070.1)	100%	Actinobacteria
Yifu122	KG-WX	*Williamsia muralis* 3541 (JN180186.1)	99%	Actinobacteria
Yifu126	KG-LTW	*Brevibacterium halotolerans* IHB B 18060 (KU605238.1)	100%	Firmicutes
Yifu128	KG-WYH	*Streptomyces pactum* CW (KX372557.1)	99%	Actinobacteria
HMU4	YA-AB	*Streptomyces lividans* T38 (KU317912.1)	100%	Actinobacteria
HMU9	YA-HSJ	*Streptomyces sampsonii* C.P.68 (KF991641.1)	99%	Actinobacteria
BI87	YA-ZSY	*Streptomyces carpaticus* BTSS-501 (HQ711933.1)	99%	Actinobacteria
HMU15	YA-TZL	*Nocardia harenae* YIM 131100 (KX502989.1)	99%	Actinobacteria
HMU25	YA-LTY	*Streptomyces lividans* T38 (KU317912.1)	100%	Actinobacteria
HMU27	YA-CPP	*Saccharothrix texasensis* 3536 (JN180184.1)	99%	Actinobacteria
HMU29	YA-CH	*Streptomyces sampsonii* GACK10 (KP970678.1)	100%	Actinobacteria
HMU31	YA-WQY	*Streptomyces sampsonii* GACK10 (KP970678.1)	99%	Actinobacteria
HMU32	YA-WQY	*Streptomyces lividans* T38 (KU317912.1)	100%	Actinobacteria
HMU37	YA-FC	*Streptomyces violascens* YJ-R42 (KF876851.1)	100%	Actinobacteria
HMU39	YA-ZS	*Streptomyces fimicarius* 14-5 (KJ571079.1)	99%	Actinobacteria
HMU63	YA-LHM	*Rhodococcus fascians* YIM 131099 (KX502988.1)	100%	Actinobacteria
HMU64	YA-LHM	*Rhodococcus cercidiphylli* YIM 131061 (KX502977.1)	100%	Actinobacteria
HMU71	YA-DYHN	*Streptococcus pyogenes* Sp15 (FJ662831.1)	99%	Firmicutes
HMU83	YA-WSL	*Streptomyces plumbiresistens* XB-2S-3 (JF439616.1)	100%	Actinobacteria
HMU87	YA-JHN	*Streptomyces griseoplanus* BCCO 10_1190 (KP718551.1)	100%	Actinobacteria
HMU101	YA-SYH	*Streptomyces lavendulae* xsd08101 (FJ481058.1)	100%	Actinobacteria
HMU103	YA-SYH	*Streptomyces zaomyceticus* HBUM174505 (EU841617.1)	100%	Actinobacteria
HMU105	YA-SYH	*Streptomyces albidoflavus* IHBA 9992 (KR085950.1)	99%	Actinobacteria
HMU121	YA-SYH	*Streptomyces longwoodensis* NBRC 14251 (NR_041161.1)	99%	Actinobacteria
HMU122	YA-SYH	*Streptomyces lavendulae* F1 (KU324431.1)	100%	Actinobacteria
AD05	YA-AR	*Streptomyces lividans* T38 (KU317912.1)	100%	Actinobacteria

### Broad ranges of cancer types to attack: differential efficacies

As most anti-malignancy bacterial strains exhibited broad ranges of cancer types to attack (see Figure 2), we focused on two selected strains, AD05 and BI87, to further investigate their spectra of anti-malignancy activities.

**Figure 5:**
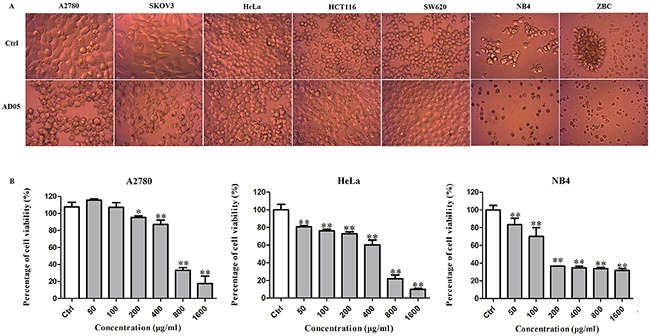
Anti-cancer activities of AD05 culture against human cancer cell lines **(A)** Morphology of different cancer cell lines after AD05 treatment. **(B)** Dose dependence of anti-cancer effects of AD05 culture extract on cervical cancer cell line HeLa, ovarian cancer cell line A2780 and human acute promyelocytic leukemia M3 cell line NB4 estimated by MTT assays. Cells were treated with various concentrations of AD05 (0, 50, 100, 200, 400, 800 and 1600 μg/ml for 24h. Data were presented as mean ± standard deviation (SD) of at least three independent experiments. *P<0.05, **P<0.01 *versus* control.

AD05 had exceptionally strong anti-malignancy activities and high stability of the effects compared to many other strains. Although most of the 76 bacterial strains exhibited strong anti-malignancy effects only on solid cancer types, AD05 exerted as strong anti-malignancy effects on leukemia as on solid cancers. We extracted the supernatant of AD05 culture with ethyl acetate and re-dissolved the dried matter in the medium. Similar to the AD05 culture supernatant, the ethyl acetate extract of AD05 also showed broad anti-cancer effects on cancer types such as ovarian cancer (A2780 and SKOV3), cervical cancer (HeLa), colorectal cancer (HCT116, SW620), human acute promyelocytic leukemia M3 (NB4), as well as a primary culture of freshly isolated chronic lymphocytic leukemia strain (ZBC) (Figure 5A). In order to examine the growth inhibitory activity of the AD05 extract on different human cancer cell lines, we used MTT assay to measure the cell viability after treatment with different concentrations. The AD05 extract exhibited significant growth inhibitory activity (P<0.05) against all the cell types tested in a dose dependent manner (Figure 5B).

**Figure 6:**
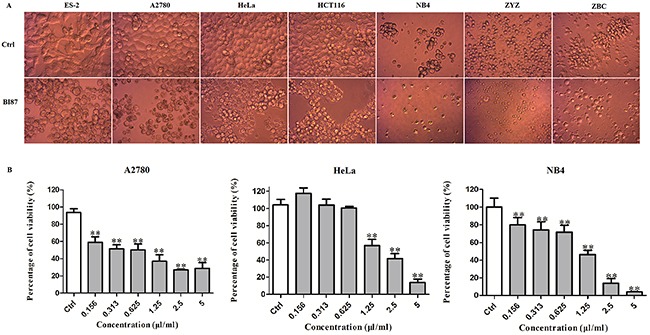
Anticancer activities of BI87 culture against human cancer cell lines **(A)** Morphology of different cancer cell lines after BI87 treatment. **(B)** Dose dependence of anti-cancer effects of BI87 culture on cervical cancer cell line HeLa, ovarian cancer cell line A2780 and human acute promyelocytic leukemia M3 cell line NB4 estimated by MTT assays. Cells were treated with the various concentrations of BI87 (0, 0.156, 0.313, 0.625, 1.25, 2.5 and 5 μl/ml) for 24h. Data were presented as mean ± standard deviation (SD) of at least three independent experiments. *P<0.05, **P<0.01 *versus* control.

Strain BI87 also exhibited a broad killing spectrum of cancers including ovarian cancer (ES2 and A2780), cervical cancer (HeLa), colorectal cancer (HCT116), leukemia (NB4), primary cultures of a freshly isolated acute monocytic leukemia M5 strain ZYZ and of a chronic lymphocytic leukemia strain ZBC (Figure 6A), and the MTT assays demonstrated that the effects were dose dependent (Figure 6B).

### BI87 suppressed cell proliferation by inducing cell apoptosis

**Figure 7:**
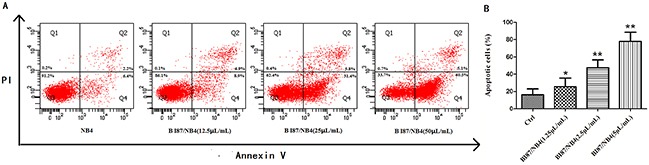
BI87 culture induced cancer cells apoptosis **(A)** The apoptosis induced by BI87 culture detected by flow cytometry. **(B)** Quantitative analysis of apoptotic cells (*P<0.05, **P<0.01 versus control).

To determine the possible mechanism of the anti-cancer effects of BI87 and AD05, we detected the induction of apoptosis after treatment. Six hours after treatment with different concentrations, cells were double stained with Annexin V and PI and subjected to flow cytometry to quantitatively analyze the apoptotic effects. As illustrated in Figure 7, the percentages of total apoptotic cells, including the early apoptotic portion (Annexin V positive) and the late apoptotic portion (Annexin V and PI positive), were dose-dependently increased with increasing concentrations of BI87 in leukemia cell lines. Under the same experiment conditions, no significant differences were detected between the AD05 culture-treated group and the controls (data not shown). These results suggest that the BI87 but not AD05 culture could suppress cell proliferation by inducing cell apoptosis.

### AD05 and BI87 inhibited both growth and metastasis of ovarian cancer *in vivo*

**Figure 8:**
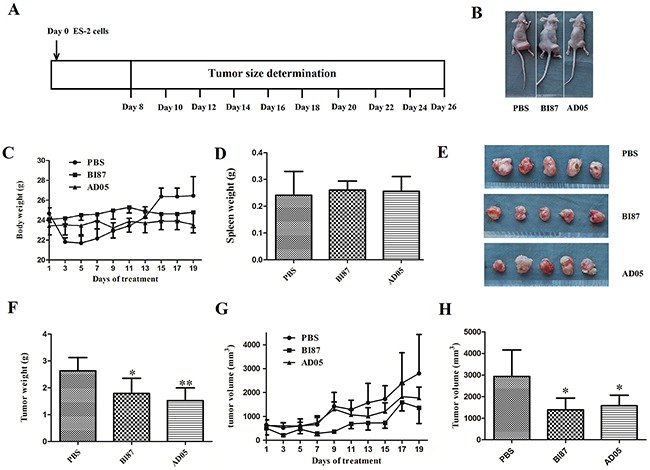
Tumor growth suppression *in vivo* by the cultures of BI87 and AD05 **(A)** Cancer cell inoculation and tumor size measurement schedule. **(B)** The subcutaneous xenograft tumor models by transplanting human ovarian cancer ES-2 cells into nude mice. **(C)** Body weight changes over the 19 days of tumor growth. **(D)** Statistical comparison of spleen weight among the animals of the three groups. The data represent the means±SD. No significant differences were seen between BI87/AD05 treated animals and controls. The *P* values were 0.6644/0.7540 respectively. **(E)** Tumor growth at day 26 in the BI87- and AD05-treated and PBS groups. **(F)** The removed tumors were weighted and statistically analyzed (n=5). The data represent the means ±SD. *P<0.05, **P<0.01 versus control. **(G)** Tumor volume changes over the 19 days of tumor growth. **(H)** Tumor volume comparison among the animals of the three groups at day 26. The data represent the means ±SD. *P<0.05 versus control.

To test the anti-cancer effects of AD05 and BI87 *in vivo*, we generated a subcutaneous xenograft tumor model by transplanting ovarian cancer ES-2 cells into nude mice (Figure 8A and 8B). AD05 and BI87 treatment as single agent resulted in significant tumor volume and weight reduction compared to treatment with PBS as control (Figure 8E, 8F, 8G and 8H). The inhibition rate of tumor weight in AD05 group was 42.16%, which was a little higher than that in the BI87 group (32.02%; Figure 8F). Of great importance, no significant differences were found in spleen or body weight among the mice (Figure 8C, 8D), indicating that there was little or no harm of the anti-cancer compounds to the general health status of the mice.

**Figure 9:**
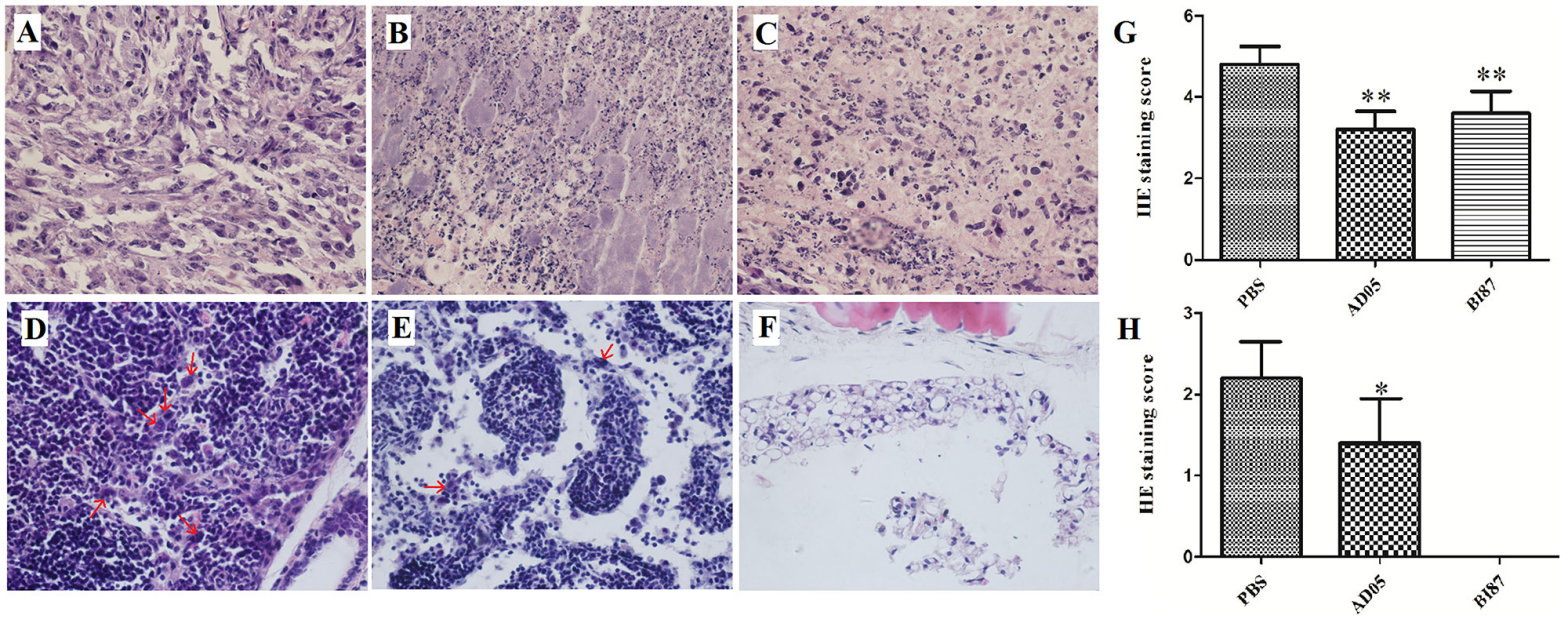
Histological examination of metastasis of cancer cells in nude mice with or without treatment (HE stain; magnification×400) Sections of tumor tissues from mice treated with PBS **(A)**, AD05 **(B)** or BI87 **(C)**, and sections of tissues on opposite side (without tumor growth by naked eyes) from mice treated with PBS **(D)**, AD05 **(E)** or BI87 **(F)**. **(G)** The statistical analyses of the HE staining score for the right thigh. **(H)** The statistical analyses of the HE staining score for the left thigh. The score 0 to 5 used here depended on the percentages of cancer cells stained by HE (0: 0%, 1: <5%, 2: 5%-25%, 3: 25%-50%, 4: 50%-75% and 5: >75%). It was determined by checking 15slides under the microscope (×400). The score for each slide was calculated from 5 different vision fields randomly.

Histological examinations of the tumor tissues revealed remarkable differences in the cancer cell morphology between mice of the experimental and control groups. Specifically, several necrotic foci with pyknotic and fragmented cell nuclei were observed in sections derived from BI87/AD05-injected tumors but not in sections derived from the controls, demonstrating that the supernatant of BI87/AD05 induced cell death (Figure 9A, B, C).

As metastasis is a common cause of cancer exacerbation and patient death, we looked into the possible effects of AD05 and BI87 on the migration or survival of cancer cells at remote sites from the primary tumor by examining legs on the opposite side (the tumor was inoculated and grew in the right thigh and tissue was taken from the left thigh where no tumor growth was seen for metastasis examination). The animal experiments showed sharp contrast between AD05- or BI87-treated and control mice. In the control, the interstitial tissue of the left thigh was heavily infiltrated with cancer cells (Figure 9D) even though no tumor growth was seen by naked eyes; in AD05-treated mice, infiltrated cancer cells were only seldom found (Figure 9E); and in BI87-treated mice, no infiltrated cancer cells were found (Figure 9F). These results demonstrated that AD05 and BI87 cultures could not only kill cancer cells but also prevent tumor metastasis effectively, with BI87 having higher performance than AD05. The statistical analyses of the HE staining scores also confirmed these effects (Figure 9G, H).

### GC-MS analysis of BI87 extract

**Figure 10:**
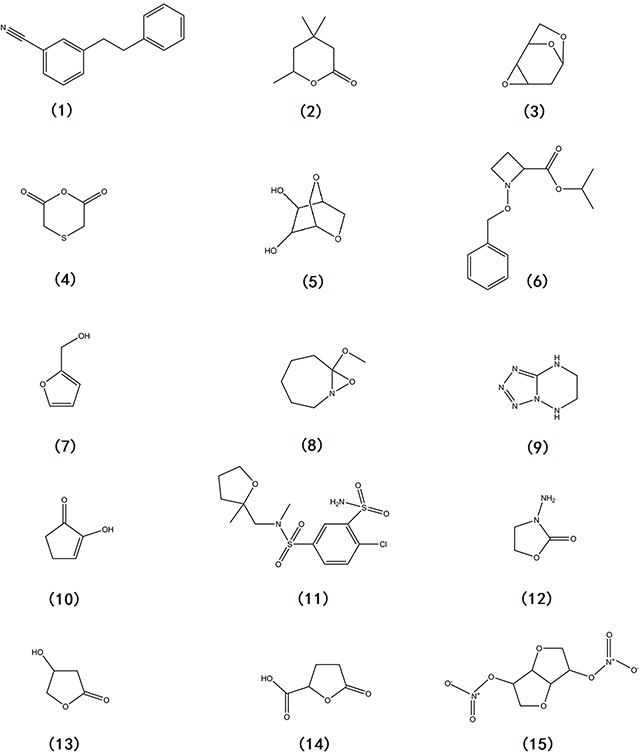
Chemical structures of constituents detected in BI87 extract

Gas chromatography-mass spectrometry analysis identified three compounds present in the ethyl acetate extract of BI87 original sample (Table 3), including (1) 3-Phenethylbenzonitrile, (2) Tetrahydro-4,4,6-trimethyl-2H-pyran-2-one, and (3) 1,6:3,4-Dianhydro-2-deoxy-β-d-ribo-hexopyranose (Figure 10). Additionally, twelve compounds were identified in the methanol extract of BI87 baked sample (Table 3), including (4) Thiodiglycolic anhydride, (5) Dianhydromannitol, (6) N-Benzyloxy-2-isopropoxycarbonylazetidine, (7) Furfuryl alcohol, (8) 7-Methoxy-8-oxa-1-arabicycle(5,1,0)octane, (9) [1,2,3,4]]Tetrazolo[1,5-b][1,2,4]triazine,5,6,7,8-tetrahydro-, (10) 2-Hydroxy-2-cyclopenten-1-one, (11) Mefruside, (12) 3-Amino-2-oxazolidinone, (13) (+/−)-3-Hydroxy-gamma-butyrolactone, (14) 2-Furancarboxylic acid, tetrahydro-5-oxo-, and (15) Isosorbide dinitrate (Figure 10). These compounds were among the most abundant components in the bacterial culture supernatant, although their involvement in the anti-malignancy activities awaits further investigations.

**Table 3:** Chemical constituents identified in *Streptomyces sp*. BI87 extract

No.	Constituents	Class	Retention time (min)	Molecular formula	Mol. weight	References
1	3-Phenethylbenzonitrile	Benzonitrile	4.191	C_15_H_13_N	207	Molander, Gary A. et al., 2002
2	Tetrahydro-4,4,6-trimethyl-2H-pyran-2-one	Furan	10.191	C_8_H_14_O_2_	142	Filip Boratynski et al., 2010
3	1,6:3,4-Dianhydro-2-deoxy-β-d-ribo-hexopyranose	Pytanose	18.478	C_6_H_8_O_3_	128	Crandall, Jack K.et al., 1983
4	Thiodiglycolic anhydride	Anhydride	1.948	C_4_H_4_O_3_S	132	Dar'in, Dmitry et al., 2015
5	Dianhydromannitol	Mannitol	2.551	C_6_H_10_O_4_	146	No paper
6	N-Benzyloxy-2-isopropoxycarbonylazetidine	Azetidine	5.233	C_14_H_19_NO_3_	249	Kostyanovskii, R.G.et al., 1974
7	Furfuryl alcohol	Furan	7.382	C_5_H_6_O_2_	98	Nicolas Bosq et al.,2015
8	7-Methoxy-8-oxa-1-arabicycle(5,1,0)octane	Azepane	8.056	C_7_H_13_NO_2_	143	Donald H. Aue et al., 1974
9	[1,2,3,4]]Tetrazolo[1,5-b][1,2,4]triazine, 5,6,7,8-tetrahydro-	Tetrazolo	8.986	C_3_H_6_N_6_	126	Rodney L.Willer et al., 1987
10	2-Hydroxy-2-cyclopenten-1-one	Cyclopentene	9.153	C_5_H_6_O_2_	98	W. Kreiser et al., 1996
11	Mefruside	Sulfonamides	10.166	C_13_H_19_ClN_2_O_5_S_2_	382	Perst V. et al., 2002
12	3-Amino-2-oxazolidinone	Oxazolidinone	13.246	C_3_H_6_N_2_O_2_	102	Gregory K. Friestad et al., 2000
13	(+/−)-3-Hydroxy-gamma-butyrolactone	Butyrolactone	13.669	C_4_H_6_O_3_	102	Perosa A. et al., 2002
14	2-Furancarboxylic acid, tetrahydro-5-oxo-	Furancarboxylic acid	14.529	C_5_H_6_O_4_	130	Montagnat.Oliver D. et al., 2010
15	Isosorbide dinitrate	Isosorbide	15.003	C_6_H_8_N_2_O_8_	236	McComb MN et al., 2016

## DISCUSSION

To our knowledge, it is the first report demonstrating the widespread existence of phylogenetically diverse bacteria that produce cancer-killing substances in the human intestine. Of particular significance, the cancer-killers have broad spectra of malignancy types to act on, including solid tumors as well as leukemia. This finding reflects a very important possibility that the anti-malignancy compounds might work not only locally (i.e., against colorectal cancers) but also systemically (i.e., against cancers in organs not in physical vicinity with the bacteria that produce the anti-malignancy compounds, such as the bone marrow, uterus, liver, ovaries, breasts, etc.), clearing stochastically appearing malignant cells at the originating site before they form lumps (solid tumors) or spreading to the whole body (leukemia and metastatic solid cancer cells) at very early stages. To date, however, investigations about bacteria and cancer have focused largely on the bacterial roles in causing (rather than clearing) cancers, especially locally, such as the well-known examples of gut bacteria causing colorectal cancer (see excellent recent reviews) [18–20]. Anti-malignancy microbes have been described mostly on viruses [21, 22], but very few on bacteria [23]. The great phylogenetic diversity of the bacteria to produce anti-malignancy substances and their broad ranges of cancer types to target demonstrate the potential clinical impacts of such bacteria and their products on malignancy therapeutics.

Among all known bacteria, actinobacteria are the best producers of bioactive secondary metabolites, producing more than two-thirds of all naturally derived antibiotics in current clinical use, as well as many anticancer, antihelmintic, and antifungal compounds [24]. Actinomycetes are closely related to the production and life of human beings. They are widely distributed in nature, and isolates from soils and oceans have so far been very extensively investigated. According to recent reports, more than 95% of the bacteria in the intestinal microbiota can be assigned to one of four major phyla: Firmicutes, Bacteroidetes, Actinobacteria and Protecteobacteria [25]. In the present study, we found that most of the cancer-killing bacteria that we had identified are actinomycetes, which might be a novel source to explore new active compounds for drug discovery.

A particularly important finding in this study is that the preschool children population seemed to have a higher ratio of individuals to possess the malignancy-killing activities than the older population (see Table 1), suggesting a fading trend of the anti-malignancy activities with age. The fact that we isolated cancer-killing bacteria also from fecal specimens tested negative for the anti-malignancy activities in the fresh supernatant indicates that the malignancy-killers might be even more widespread in the human populations than shown here but the eventual expression of their malignancy-killing effects might require optimal balancing and coordination of multiple factors, which may involve human genetic predisposition as well as environmental disturbance such as dysbiosis due to certain kinds of diets or abusive use of antimicrobials, which calls for further investigation.

An exceptionally interesting fact is that one individual may have phylogenetically very diverse malignancy-killers, as seen in participant KG-HYX, who had the malignancy-killers belonging to two phyla, Firmicutes and Actinobacteria; within a phylum, the diversity was also enormous (see Table 2 and Supplementary Figure 2). It can be anticipated that larger scales of screening of the intestinal microbes, not only bacteria but possibly also other kinds of microorganisms like fungi or even protozoa, may reveal even greater diversity of malignancy-killers. As such, mechanisms used by the malignancy-killers for the anti-cancer activities may involve extensive molecular pathways.

Our findings in this study bring up four updates to the biomedical research fields, especially regarding carcinogenesis investigations and clinical therapeutics of cancers. First, the widespread existence of malignancy-killers in the human populations at large provides a novel explanation to the fact that many people at high genetic or environmental risks to cancer may not have cancer in their long lifetime, revealing a long ignored arsenal against cancers in addition to the immune system. Second, the importance of maintaining a normal microbiota, such as by avoiding antimicrobial abuse, is reiterated to reduce cancer risks. Third, as the isolated bacterial strains that produced the anti-malignancy activities are phylogenetically very diverse and the damages caused by the cancer-killing effects of the bacteria to the cancer cells are morphologically remarkably distinct, the anti-malignancy substances produced by the bacteria, and hence the mechanisms in cancer killing, may be highly different, encouraging the combined use of the different substances for even greater effects in the treatment of cancers. Finally, the natural anti-cancer substances may become novel lead compounds for cancer drug development.

## MATERIALS AND METHODS

### Collection of fecal specimens and isolation of bacteria

Fecal specimens were collected from 100 preschool children of Yifu Kindergarten, 3-7 years old, and 113 young adults, 18-39 years old, including faculty, postdoctoral fellows, and graduate and undergraduate students, Harbin Medical University, Harbin, China. We obtained the informed consents and detailed information of the participants including age, sex, and family history. The fecal specimens were mixed with equal volume of sterile distilled water and centrifuged at 10000 rpm/min for 30 min. The supernatant was saved for detection of anti-cancer effects and the pellets were treated as follows for bacterial isolation. One gram of the fecal pellet was suspended in 10 ml PBS (KH_2_PO_4_ 0.27g, NaHPO_4_ 1.42g, NaCl 8g, KCl 0.2g and 1L distilled water) and shaken for 30 minutes at 60 rpm/min. After the shaking, we placed the tubes on the bench and waited for 10 min for the large debris to become settled to the bottom of the tubes. We then inoculated the upper part of the samples by to 10:1, 100:1 and 1000:1 dilutions into the Gauze No. 1 medium (solution starch 20 g, KNO_3_ 1 g, K_2_HPO_4_ 0.5g, MgSO_4_·7H_2_O 0.5g, NaCl 0.5g, FeSO_4_·7H_2_O 0.01g, K_2_Cr_2_O_7_ 50mg and distilled water added to 1L) and incubated the mixture at 28°C. Periodically, we spread the liquid culture onto Gauze No. 1 agar plates and picked up colonies with different morphological characteristics, with a focus on actinobacteria-like bacteria. The chosen colonies were transferred into fresh Gauze No.1 liquid medium for further culture and saturated pure cultures were obtained for tests as well as for preservation in 25% (v/v) glycerol at -80°C.

### Cell culture

Human solid cancer cells of several cell lines, including cervical cancer cell line HeLa, ovarian cancer cell lines SKOV3 and A2780, and colorectal cancer cell lines HCT116 and SW620, were cultured in Dulbecco's modified Eagle's medium (DMEM) with 10% fetal bovine serum. Ovarian cancer cell line ES-2 was cultured in McCoy's 5A medium with 10% fetal bovine serum. Human acute promyelocytic leukemia NB4 cells were cultured in RPMI 1640 medium, supplemented with 10% fetal bovine serum, 100 units/mL penicillin and 100 μg/mL streptomycin. All the cultures were maintained in an incubator at 37°C with 5% CO_2_ in a humidified atmosphere.

### Isolation and cultivation of leukemia cells

Heparin-anticoagulated peripheral blood from a leukemia patient was centrifuged at 1500 rpm/min for 10 min. The plasma was removed and the blood cells were mixed with equal volume of cell culture medium without serum. The mixture was then added slowly to the top of two volumes of leukocyte separation medium (Tianjin Haoyang biological technology co., LTD) before centrifugation at 2000 rpm/min for 20 min. The leukemia cells in the leukocyte layer were transferred to a fresh 15 ml sterile centrifuge tube, washed with PBS and cultured in RPMI 1640 medium supplemented with 10% fetal calf serum in an incubator at 37°C with 5% CO_2_ in a humidified atmosphere.

### Morphological assessment

Morphological changes of cells treated with fecal supernatant or extractions of the metabolites from the bacterial cultures were inspected by phase contrast inverted microscopy (Zeiss Axiocam Erc 5s, Germany). The performance of the experiments and the determination of experimental results were completed blindly and separately by at least two different persons. The cells treated with the same volume of Gauze No.1 medium and the cells without treatment served as control groups.

### Bacterial genomic DNA extraction

Genomic DNA from bacterial strains that showed strong anti-cancer activities was isolated for 16S rDNA sequencing. For each strain, we picked a single colony and inoculated it to 5 mL Gauze No.1 medium. The incubation was conducted in a shaker at 28°C for 7 days. After that, we transferred the culture to 50 mL Gauze No.1 fresh medium to continue the incubation for 7 days. After centrifugation at 4000 rpm/min for 15 min, the supernatant was discarded. The pellets were crushed down and suspended in 5 ml 10 mM EDTA and then the suspension was centrifuged at 4000 rpm/min for 15min. Next, the pellet was suspended in 5 ml SET buffer (10 ml 1M Tris-HCl pH7.5, 25ml 0.5M EDTA pH8.0, 7.5 ml 5M NaCl, and H_2_O adjusted to 1L). Following that, we added 50 μl 100 mg/ml lysozyme to the suspension and kept it at 37°C for 1 hour, and then added 600 μl 10% SDS and 150 μl 20mg/ml proteinase K to the suspension and kept it at 55°C for 2 hours. After mixing the suspension with 2 ml 5M NaCl and 5 ml chloroform and shaking the mixture for 30 minute, we centrifuged it at 4000 rpm/min for 30 min and transferred the aqueous phase to an Eppendorf tube. To this, we added an equal volume of isopropanol and centrifuged the tube at 8000 rpm/min for 20 min. We washed the DNA pellet by adding 5 ml 70% ethanol and centrifuging the tube at 8000 rpm/min for 20 min. After the DNA pellet was air dried, we added 200 μl TE buffer to it and stored it at −20°C.

### 16S rRNA gene amplification and phylogenetic analyses

Primers for amplifying the 16S rRNA gene were designed according to the highly conserved regions of 16S rRNA gene sequences [14], and we used F (5′-AGAGTTTGATCCTGGCTCAG-3′) and R (5′-AAGGAGGTGATCCAGCCGCA-3′) here. The 25 μl PCR mixture contained 1 μl bacterial genomic DNA, 1 μl primer F and 1 μl primer R, 0.5 μl dNTP, 0.2 μl DNA polyase, 2 μl PCR buffer and 19.3 μl deionized water. The PCR conditions were as follows: the initial denaturation was set at 95°C for 5 min, followed by 10 cycles of 94°C for 30 s, 61°C for 30 s, and 72°C for 45 s, then 25 cycles of 94°C for 30 s, 53°C for 30 s, and 72°C for 45 s, with final extension for 10 min at 72°C. All amplification products were sent to Genewiz company for sequencing. The 16S rDNA sequence data reported in the present study were deposited in the Genbank nucleotide database under the accession numbers KX900586 (Yifu7), KU058402 (Yifu8), KU058420 (Yifu20), KU058403 (Yifu38), KX900587 (Yifu52), KX900588 (Yifu60), KX900589 (Yifu61), KU058404 (Yifu62), KU058414 (Yifu63), KX900590 (Yifu65), KU058415 (Yifu68), KX900591 (Yifu69), KU058405 (Yifu75), KU058416 (Yifu78), KU058417 (Yifu80), KX900592 (Yifu88), KX900593 (Yifu89), KU058418 (Yifu91), KX900594 (Yifu95), KU058406 (Yifu100), KU058419 (Yifu104), KX900595 (Yifu105), KX900596 (Yifu113), KX900597 (Yifu115), KX900598 (Yifu116), KX900599 (Yifu117), KX900600 (Yifu120), KX900601 (Yifu121), KX900602 (Yifu122), KX900603 (Yifu126), KX900604 (Yifu128), KU058421 (HMU4), KX900573 (HMU9), KU058407 (BI87), KX900574 (HMU15), KX900575 (HMU25), KU058408 (HMU27), KX900576 (HMU29), KX900577 (HMU31), KX900578 (HMU32), KX900579 (HMU37), KU058409 (HMU39), KX900580 (HMU63), KX900581 (HMU64), KU058410 (HMU71), KX900582 (HMU83), KX900583 (HMU87), KU058411 (HMU101), KU058412 (HMU103), KU058413 (HMU105), KX900584 (HMU121), KX900585 (HMU122), KU058430 (AD05). The sequences were compared for similarity with the reference species of bacteria using the NCBI BLAST. They were aligned using the Clustal X program [15] and the phylogenetic tree was constructed by MEGA 6 using the neighbor-joining algorithm [16]. Evolutionary distances for the neighbor-joining algorithm were computed using p-distance model. The stability of the tree topologies were evaluated by using the bootstrap based on 100 replications.

### Extraction of the metabolites from bacterial cultures

The culture supernatant was filtered by vacuum and then extracted by equal volume of ethyl acetate for three times. The extraction was dried by rotary evaporator at 40°C and stored at 4°C.

### MTT assay

Cultured cells were distributed into 96-well plates in 100 μL of medium, incubated overnight and then treated with the compounds over a range of concentrations for the indicated time. After the addition of 20 μL of 5 mg/mL MTT solution per well, the plates were incubated for another 4 h. The media were removed, and the formazan crystals were solubilized in 150 μL of DMSO and shaken for 10 min. The absorbance was measured at 490 nm using a microplate reader (Emax@ Plus, England). The percentage of cell viability was calculated as follows:

% cell viability = Absorbance of treated cells/Absorbance of untreated cells × 100%

### Cell apoptosis analysis

To detect apoptosis, cells were incubated with culture of BI87 or AD05 for 6h. The cells were harvested, washed twice with cold 1xPBS, and re-suspended in 100μL 1×Binding buffer at density of 1×10^5^ cells/mL. The cells were then stained with 5μL Annexin V and 5μL PI (BD Biosciences) for 15min in dark condition at room temperature. After staining, we added 400 μl of 1×Binding Buffer to each tube. The samples were subjected to analysis by flow cytometry (BD FACSCanto^TM^ II). The early apoptosis was evaluated based on the percentage of Annexin V positive and PI negative cells, while the late apoptosis was evaluated based on the percentage of Annexin V positive and PI positive cells.

### *In vivo* tumor growth assay

Female nude mice at 6-8 weeks of age were purchased from Beijing Vital River Laboratory Animal Technology Co., Ltd., and housed in a specific pathogen free facility. Mice were subcutaneously inoculated with ES-2 cells (0.33 × 10^6^ suspended in 0.08 mL PBS for each mouse). When the tumor reached an average volume of 500 mm^3^, the animals were randomized into groups (n=5) and treated with sterile supernatant of the bacterial cultures or with PBS, 1ml/kg, by intra-tumor injection every other day. The measurements of tumor growth with a digital caliper were done once every other day. Tumor volumes were calculated by the two dimensional sizes of each tumor with the following formula: V = L×W^2^×1/2, where V is the volume, L is the length, and W is the width. At the end of experiment, the mice were weighed and sacrificed, and the tumors were dissected, weighed and stored in 4% paraformaldehyde solution for hematoxylin-eosin staining.

### Hematoxylin-eosin staining

Following euthanasia of the mice, the tumor tissue was removed aseptically and immediately fixed in a 4% paraformaldehyde solution (Wuhan Boster Biological Technology Co., Ltd). The fixed tumors were processed through graded concentrations of ethanol and xylene and were then embedded in paraffin. The tissue sections were mounted on glass slides and stained with HE. In total, 15 slides were inspected and the scoring was performed.

### Gas chromatography-mass spectrometry (GC-MS) analysis

We employed the Agilent Technologies 7890B (GC) machinery equipped with 5977A Mass Selective Detector (MS), HP-5MS (5% phenyl methyl siloxane) capillary column of dimensions 30m×250μm×0.25μm and used helium as the carrier gas at 1 mL/min. For the original sample, the column temperature was programmed initially at 40°C for 3min, followed by an increase of 10°C /min to 220°C and then an increase of 25°C /min to 280°C. For the baked sample, the column temperature was programmed initially at 40°C for 5min, followed by an increase of 10°C /min to 180°C and then an increase of 20°C /min to 280°C. The MS was operated at 70eV. The constituents were identified by comparison of their mass spectral data with those from NIST 11 Spectral Library.

### Statistical analysis

Data were presented as mean ± standard deviation (SD) of at least three independent experiments. The MTT assay results were obtained by GraphPad Prism statistical software. The statistical significance was accepted at P < 0.05.

## SUPPLEMENTARY FIGURES AND TABLE




