# Supplementary material for: Cancer killers in the human gut microbiota: diverse phylogeny and broad spectra
Source: Oncotarget. 2017 Apr 21;8(30):49574–91. doi: 10.18632/oncotarget.17319 (PMC5564789; doi:10.18632/oncotarget.17319)
Supplement: Supplementary file 1 [file oncotarget-08-49574-s001.pdf]

Cancer killers in the human gut microbiota: diverse phylogeny and broad spectra

SUPPLEMENTARY FIGURES AND TABLE

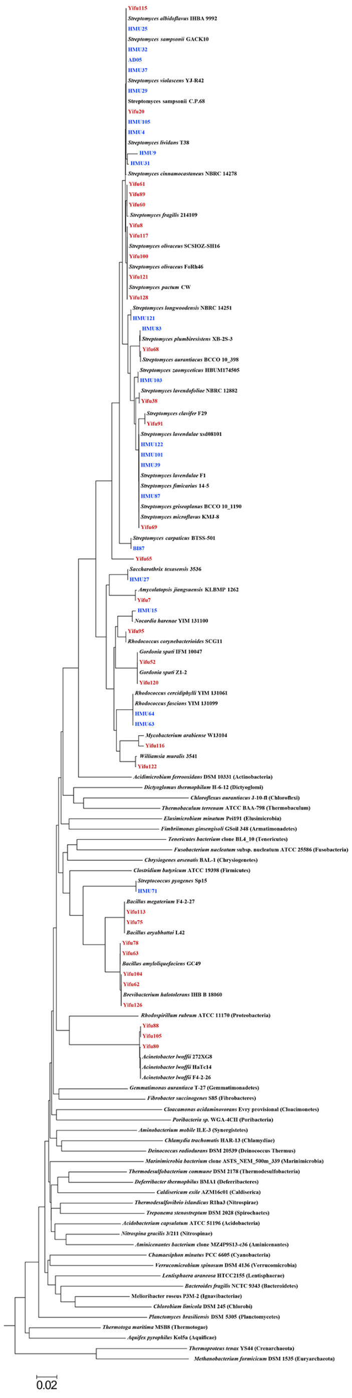

Supplementary Figure 1: Phylogenetic tree derived from 16S rRNA gene sequence analysis. The 53 bacterial strains with strong anticancer activities are shown in color. The tree was generated by Neighbor-Joining method.

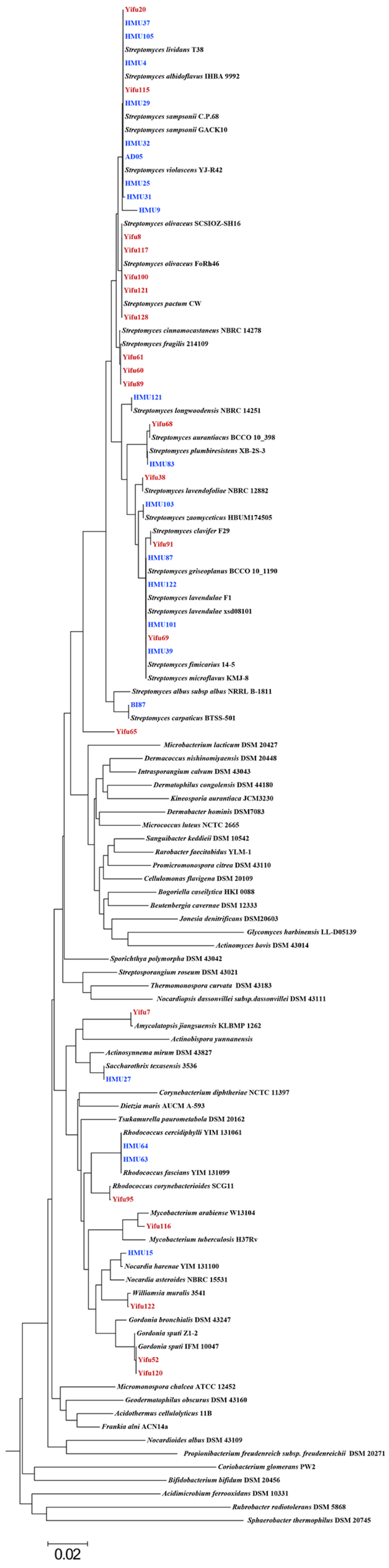

Supplementary Figure 2: Phylogenetic tree derived from 16S rRNA gene sequence analysis. The 42 Actinomyces strains with strong anticancer activities are shown in color. The tree was generated by Neighbor-Joining method.

Supplementary Table 1: Participants and the anti-cancer activities of their fecal specimens.

See Supplementary File 1
