# Supplementary material for: Cancer killers in the human gut microbiota: diverse phylogeny and broad spectra
Source: Oncotarget. 2017 Apr 21;8(30):49574–91. doi: 10.18632/oncotarget.17319 (PMC5564789; doi:10.18632/oncotarget.17319)
Supplement: Supplementary file 2 [file oncotarget-08-49574-s002.docx]

**Supplementary Table 1**. Participants and the anti-cancer activities of their fecal specimens.

| **No.** | **Participant ID** | **Sex** | **Age** | **Activity** | **Strains with anti-cancer activity** |
| --- | --- | --- | --- | --- | --- |
| 1 | KG-YRK | male | 5 | ＋ |  |
| 2 | KG-FYZ | male | 5 | ＋ |  |
| 3 | KG-WZW | male | 5 | ＋ |  |
| 4 | KG-ZYC | male | 5 | ＋ |  |
| 5 | KG-LYX | female | 6 | ＋ |  |
| 6 | KG-WSC | male | 6 | ＋ |  |
| 7 | KG-JZH | female | 5 | ＋ | Yifu3、Yifu4、Yifu5、Yifu6、Yifu7*、Yifu8*、Yifu9 |
| 8 | KG-XZM | male | 6 | ＋ |  |
| 9 | KG-SDA | male | 5 | ＋ |  |
| 10 | KG-XHR | female | 6 | ＋ |  |
| 11 | KG-SYF | male | 4 | ＋ |  |
| 12 | KG-ZJH | female | 6 | ＋ |  |
| 13 | KG-HJB | male | 5 | ＋ |  |
| 14 | KG-CYF | female | 3 | ＋ |  |
| 15 | KG-SRH | male | 3 | ＋ |  |
| 16 | KG-ZBY | male | 3 | ＋ |  |
| 17 | KG-LBW | male | 4 | ＋ |  |
| 18 | KG-WYB | female | 3 | ＋ |  |
| 19 | KG-WYH | female | 3 | ＋ | Yifu15、Yifu39、Yifu128* |
| 20 | KG-WYH | male | 5 | ＋ |  |
| 21 | KG-DYN | male | 3 | ＋ |  |
| 22 | KG-WHZ | male | 3 | ＋ |  |
| 23 | KG-GB | male | 4 | ＋ |  |
| 24 | KG-CXR | female | 5 | ＋ |  |
| 25 | KG-KK | male | 6 | ＋ |  |
| 26 | KG-XXH | female | 4 | ＋ |  |
| 27 | KG-ZHC | male | 5 | ＋ |  |
| 28 | KG-ZWH | female | 4 | ＋ |  |
| 29 | KG-CEY | female | 4 | ＋ |  |
| 30 | KG-HYX | male | 3 | ＋ | Yifu19、Yifu20* |
| 31 | KG-QMZ | male | 3 | ＋ |  |
| 32 | KG-LMQ | male | 4 | － |  |
| 33 | KG-ZHL | male | 4 | － |  |
| 34 | KG-QJY | male | 4 | － |  |
| 35 | KG-ZMC | female | 3 | － |  |
| 36 | KG-XZX | female | 3 | ＋ |  |
| S | KG-HD | female | 5 | － | Yifu47 |
| 38 | KG-DYL | female | 4 | ＋ |  |
| 39 | KG-XWR | female | 6 | － | Yifu115*、Yifu116* |
| 40 | KG-YPY | male | 5 | ＋ | Yifu114、Yifu113* |
| 41 | KG-YPY | female | 6 | － |  |
| 42 | KG-LTW | male | 5 | ＋ | Yifu126* |
| 43 | KG-SBW | male | 4 | － |  |
| 44 | KG-WLC | male | 5 | － |  |
| 45 | KG-ZAK | female | 4 | － |  |
| 46 | KG-QGW | male | 4 | － |  |
| 47 | KG-PHX | male | 5 | ＋ |  |
| 48 | KG-CPY | male | 6 | － |  |
| 49 | KG-LYR | female | 6 | － |  |
| 50 | KG-SR | female | 5 | ＋ | Yifu38* |
| 51 | KG-WJS | male | 6 | － |  |
| 52 | KG-HBY | male | 5 | ＋ | Yifu52* |
| 53 | KG-LT | male | 3 | ＋ |  |
| 54 | KG-GRY | female | 3 | － |  |
| 55 | KG-LEQ | female | 3 | － | Yifu117*、Yifu118 |
| 56 | KG-LZY | male | 4 | ＋ |  |
| 57 | KG-CYR | female | 3 | － |  |
| 58 | KG-ZYC | female | 4 | － | Yifu60*、Yifu61*、Yifu62*、Yifu63* |
| 59 | KG-XWQ | female | 4 | － |  |
| 60 | KG-WZH | male | 6 | － | Yifu65* |
| 61 | KG-JYX | male | 5 | － | Yifu68*、Yifu69* |
| 62 | KG-YCX | female | 3 | － |  |
| 63 | KG-JYX | female | 5 | ＋ |  |
| 64 | KG-ZJH | female | 3 | ＋ | Yifu75*、Yifu119、Yifu120* |
| 65 | KG-SJX | male | 5 | ＋ | Yifu78* |
| 66 | KG-LJY | female | 6 | － |  |
| 67 | KG-ZZJ | male | 5 | － |  |
| 68 | KG-LZY | female | 3 | ＋ |  |
| 69 | KG-WSY | male | 5 | － |  |
| 70 | KG-XHS | male | 4 | ＋ |  |
| 71 | KG-PJY | male | 3 | ＋ | Yifu80* |
| 72 | KG-LXZ | male | 5 | ＋ |  |
| 73 | KG-SYZ | male | 3 | － |  |
| 74 | KG-SYF | female | 6 | － |  |
| 75 | KG-FSK | female | 3 | ＋ |  |
| 76 | KG-LZT | male | 5 | ＋ |  |
| 77 | KG-WC | male | 3 | ＋ |  |
| 78 | KG-YWB | female | 5 | ＋ |  |
| 79 | KG-RHM | female | 6 | ＋ |  |
| 80 | KG-CBC | female | 4 | － | Yifu88*、Yifu89* |
| 81 | KG-HYH | female | 5 | － |  |
| 82 | KG-ZMX | female | 5 | － |  |
| 83 | KG-WX | female | 3 | － | Yifu91*、Yifu121*、Yifu122* |
| 84 | KG-YZY | female | 5 | － |  |
| 85 | KG-JYH | female | 5 | － |  |
| 86 | KG-TEZ | male | 3 | ＋ |  |
| 87 | KG-LZL | male | 5 | － |  |
| 88 | KG-WJZ | female | 3 | ＋ | Yifu95*、Yifu96、Yifu123、Yifu124 |
| 89 | KG-WYF | female | 6 | ＋ |  |
| 90 | KG-ZQ | male | 2 | － |  |
| 91 | KG-LXY | female | 4 | ＋ |  |
| 92 | KG-YQT | female | 3 | ＋ | Yifu100* |
| 93 | KG-LZR | male | 3 | ＋ |  |
| 94 | KG-HZL | male | 5 | － |  |
| 95 | KG-TZ | male | 5 | － | Yifu104* |
| 96 | KG-XZH | male | 6 | － | Yifu105*、Yifu106 |
| 97 | KG-YZY | female | 5 | ＋ |  |
| 98 | KG-ZYJ | female | 4 | － |  |
| 99 | KG-ZGX | female | 4 | － |  |
| 100 | KG-LZC | female | 3 | － |  |
| 101 | YA-YZY | female | 18 | － |  |
| 102 | YA-WB | male | 17 | － |  |
| 103 | YA-unknown | unknown | unknown | － |  |
| 104 | YA-DSJ | female | 21 | － |  |
| 105 | YA-AB | male | 19 | － | HMU4* |
| 106 | YA-TWX | male | 19 | － |  |
| 107 | YA-MP | male | 18 | － |  |
| 108 | YA-ZGQ | female | 20 | － |  |
| 109 | YA-YX | male | 20 | － |  |
| 110 | YA-WHY | female | 19 | － |  |
| 111 | YA-FCL | female | 19 | － |  |
| 112 | YA-HWM | male | 20 | － |  |
| 113 | YA-HSJ | female | 18 | － | HMU9* |
| 114 | YA-ZJL | female | 20 | － |  |
| 115 | YA-GMQ | female | 22 | － |  |
| 116 | YA-ZSY | female | 21 | － | BI87* |
| 117 | YA-SHS | female | 21 | － |  |
| 118 | YA-TZL | male | 20 | － | HMU15* |
| 119 | YA-LL | female | 20 | － |  |
| 120 | YA-SMN | female | 20 | － |  |
| 121 | YA-LY | female | 19 | － | HMU22 |
| 122 | YA-YZR | female | 20 | － |  |
| 123 | YA-LTY | female | 20 | － | HMU25* |
| 124 | YA-CPP | female | 21 | － | HMU27* |
| 125 | YA-CH | male | 21 | － | HMU29*、HMU30 |
| 126 | YA-WQY | male | 19 | － | HMU31*、HMU32* |
| 127 | YA-HQ | female | 19 | － | HMU36 |
| 128 | YA-FC | male | 22 | ＋ | HMU37* |
| 129 | YA-ZS | female | 21 | － | HMU39* |
| 130 | YA-MWH | female | 19 | － |  |
| 131 | YA-SQ | male | 22 | － | HMU45 |
| 132 | YA-LSY | female | 18 | － |  |
| 133 | YA-YXX | male | 20 | － |  |
| 134 | YA-ZYX | male | 20 | － |  |
| 135 | YA-FYJ | female | 22 | － |  |
| 136 | YA-MHY | female | 21 | － |  |
| 137 | YA-XXM | female | 19 | ＋ |  |
| 138 | YA-BJ | female | 18 | － |  |
| 139 | YA-CS | male | 21 | － |  |
| 140 | YA-SX | female | 21 | ＋ |  |
| 141 | YA-CYB | female | 21 | ＋ | HMU62 |
| 142 | YA-LHM | female | 21 | － | HMU63*、HMU64* |
| 143 | YA-LYY | female | 20 | － |  |
| 144 | YA-WRM | female | 19 | － |  |
| 145 | YA-WY | male | 19 | － |  |
| 146 | YA-LXL | female | 22 | － |  |
| 147 | YA-WYJ | female | 19 | ＋ |  |
| 148 | YA-LHC | male | 20 | － |  |
| 149 | YA-LYN | female | 21 | － |  |
| 150 | YA-SR | female | 22 | － |  |
| 151 | YA-HL | female | 21 | － |  |
| 152 | YA-DYHN | female | 21 | － | HMU71* |
| 153 | YA-CYJ | female | 22 | － | HMU75 |
| 154 | YA-WYT | female | 21 | － |  |
| 155 | YA-ZQZ | female | 22 | － |  |
| 156 | YA-SJB | male | 21 | － |  |
| 157 | YA-ZWL | male | 20 | － |  |
| 158 | YA-WHR | female | 21 | － |  |
| 159 | YA-ZYZ | male | 19 | － |  |
| 160 | YA-WJJ | female | 19 | － |  |
| 161 | YA-TJ | female | 19 | － |  |
| 162 | YA-WYY | female | 18 | － |  |
| 163 | YA-LJ | female | 19 | － |  |
| 164 | YA-SN | female | 18 | － |  |
| 165 | YA-WSL | female | 21 | － | HMU83* |
| 166 | YA-YXF | female | 19 | ＋ |  |
| 167 | YA-SH | male | 20 | － |  |
| 168 | YA-LYY | female | 20 | － |  |
| 169 | YA-JHN | male | 20 | － | HMU87* |
| 170 | YA-YMX | female | 20 | － |  |
| 171 | YA-XKL | female | 19 | － |  |
| 172 | YA-XX | female | 20 | ＋ |  |
| 173 | YA-WY | male | 20 | － |  |
| 174 | YA-YR | female | 19 | － |  |
| 175 | YA-MXL | female | 19 | － |  |
| 176 | YA-GS | female | 19 | － |  |
| 177 | YA-LZY | female | 19 | － | HMU91 |
| 178 | YA-LR | female | 19 | － |  |
| 179 | YA-SHW | female | 19 | － |  |
| 180 | YA-WB | female | 19 | － |  |
| 181 | YA-GYX | female | 21 | － | HMU119 |
| 182 | YA-WL | female | 19 | － |  |
| 183 | YA-LLJ | female | 19 | － |  |
| 184 | YA-SYH | female | 19 | － | HMU101*、HMU103*、HMU105*、HMU120、HMU121*、HMU122* |
| 185 | YA-MZY | female | 19 | － |  |
| 186 | YA-BZ | male | 18 | － |  |
| 187 | YA-FXF | female | 20 | － |  |
| 188 | YA-HJ | male | 19 | － |  |
| 189 | YA-HKP | male | 20 | － |  |
| 190 | YA-FR | female | 18 | － |  |
| 191 | YA-JT | female | 18 | － |  |
| 192 | YA-WL | female | 19 | ＋ |  |
| 193 | YA-PHM | female | 17 | － |  |
| 194 | YA-ZMM | female | 19 | ＋ |  |
| 195 | YA-SQ | male | 27 | － |  |
| 196 | YA-FX | female | 28 | － |  |
| 197 | YA-ZZY | male | 32 | － |  |
| 198 | YA-LQH | male | 36 | + |  |
| 199 | YA-LYP | female | 24 | － |  |
| 200 | YA-YZQ | female | 25 | － |  |
| 201 | YA-JG | male | 26 | － |  |
| 202 | YA-YC | male | 27 | － |  |
| 203 | YA-LBL | male | 26 | － |  |
| 204 | YA-AR | male | 39 | + | AD05* |
| 205 | YA-ZYJ | female | 28 | － |  |
| 206 | YA-LM | female | 21 | － |  |
| 207 | YA-EM | male | 39 | + |  |
| 208 | YA-ZSL | male | 37 | － |  |
| 209 | YA-ZDD | female | 24 | － |  |
| 210 | YA-LBB | female | 24 | － |  |
| 211 | YA-YL | female | 25 | － |  |
| 212 | YA-CXY | female | 24 | － |  |
| 213 | YA-ZZ | female | 22 | － |  |

* Strain with 16S rDNA sequenced (See Table 2)
